# Identification and characterization of lncRNA mediated transcriptional dysregulation dictates lncRNA roles in glioblastoma

**DOI:** 10.18632/oncotarget.7801

**Published:** 2016-03-01

**Authors:** Yongsheng Li, Zishan Wang, Yuan Wang, Zheng Zhao, Jinwen Zhang, Jianping Lu, Juan Xu, Xia Li

**Affiliations:** ^1^ College of Bioinformatics Science and Technology and Bio-Pharmaceutical Key Laboratory of Heilongjiang Province, Harbin Medical University, Harbin 150081, China

**Keywords:** long non-coding RNA, transcriptional dysregulation, lncRNA roles, lncRNA modulator, lncRNA-TF-gene triplets

## Abstract

Long non-coding RNAs (lncRNAs) modulate gene expression, and lncRNA misregulation is associated with cancer. However, precise functional roles in biological and disease processes have been described for only a few lncRNAs. Identification of genome-wide lncRNA-mediated transcriptional dysregulations may improve cancer treatments. In the present study, we used a computational framework that combined lncRNA and gene expression profiles with transcription factor (TF)-target relationships to comprehensively identify dysregulatory lncRNA-TF-gene triplets. In glioblastoma (GBM), we found that most lncRNAs affect multiple targets and primarily affect TF activity in trans. Six different classes of lncRNA-mediated transcriptional dysregulations were identified, with most lncRNAs either enhancing or attenuating target gene expression. Functional analysis of lncRNAs via their dysregulated targets implicated lncRNA modulators in some hallmarks of cancer, providing a new way to predict lncRNA function. Finally, we identified several lncRNA-TF-gene triplets (including HOTAIR-MXI1-CD58/PRKCE and HOTAIR-ATF5-NCAM1) that are associated with glioblastoma prognosis. The integration of lncRNA modulators into transcriptional regulatory networks will further enhance our understanding of lncRNA functions in cancer.

## INTRODUCTION

The eukaryotic genome harbors a large number of noncoding RNAs. In addition to well-studied small miRNAs [1], a great proportion of the transcriptome generates RNA transcripts greater than 200 nucleotides in length, which are defined as long non-coding RNAs (lncRNAs) [2]. The human genome encodes more than 15,000 potential lncRNAs according to ENCODE V23 [3]. lncRNAs interact with various biomolecules, including DNA, RNA, and proteins, to regulate gene expression at transcriptional, post-transcriptional, and epigenetic levels [4], playing important roles in a wide range of biological processes [5]. Given that lncRNAs are key regulators of gene expression, it is not surprising that they are frequently dysregulated during tumorigenesis [6, 7]. However, so far, only a few lncRNAs have been functionally linked to biological or disease processes [4]. Our knowledge of the regulatory roles of lncRNAs is limited.

Gene transcription is strictly regulated, in large part, by transcription factor (TF) proteins that bind to genomic cis-regulatory elements in a sequence-specific fashion. While TFs are the primary engines of transcription, the ability of a TF to regulate its targets is modulated by a variety of genetic and epigenetic mechanisms [8–10]. Global regulatory perturbations are also related to tumor growth and cancer progression [11, 12]. Increasing experimental evidence has shown that lncRNAs are important gene expression modulators that mediate transcriptional regulation, and lncRNA misregulation is associated with cancer. A recent study suggested that the lncRNA MALAT1 regulates E2F1 transcription factor activity, which is a crucial determinant of cell cycle progression and tumorigenesis [13]. In addition, depletion of MALAT1 activates P53 and its target genes. Furthermore, MALAT1-depleted cells display reduced expression of B-MYB, an oncogenic TF involved in the cell cycle [14]. Another lncRNA, lincRNA-p21, is also involved in cancer. LincRNA-p21, in association with hnRNP-K, represses p53-dependent transcriptional responses or suppresses target mRNA translation [15]. Moreover, the lncRNA CCAT1-L also plays a role in MYC transcriptional regulation, and CCAT1-L overexpression promoted MYC transcription and enhanced tumorigenesis [16]. These findings suggest that lncRNAs may serve as important regulators of TFs in tumorigenesis and thus comprise a new RNA-based gene regulation mechanism that complements the central dogma [17]. Therefore, studying lncRNA-mediated changes in TF activity is an important step in determining lncRNA functions at a system-wide level.

**Figure 1:**
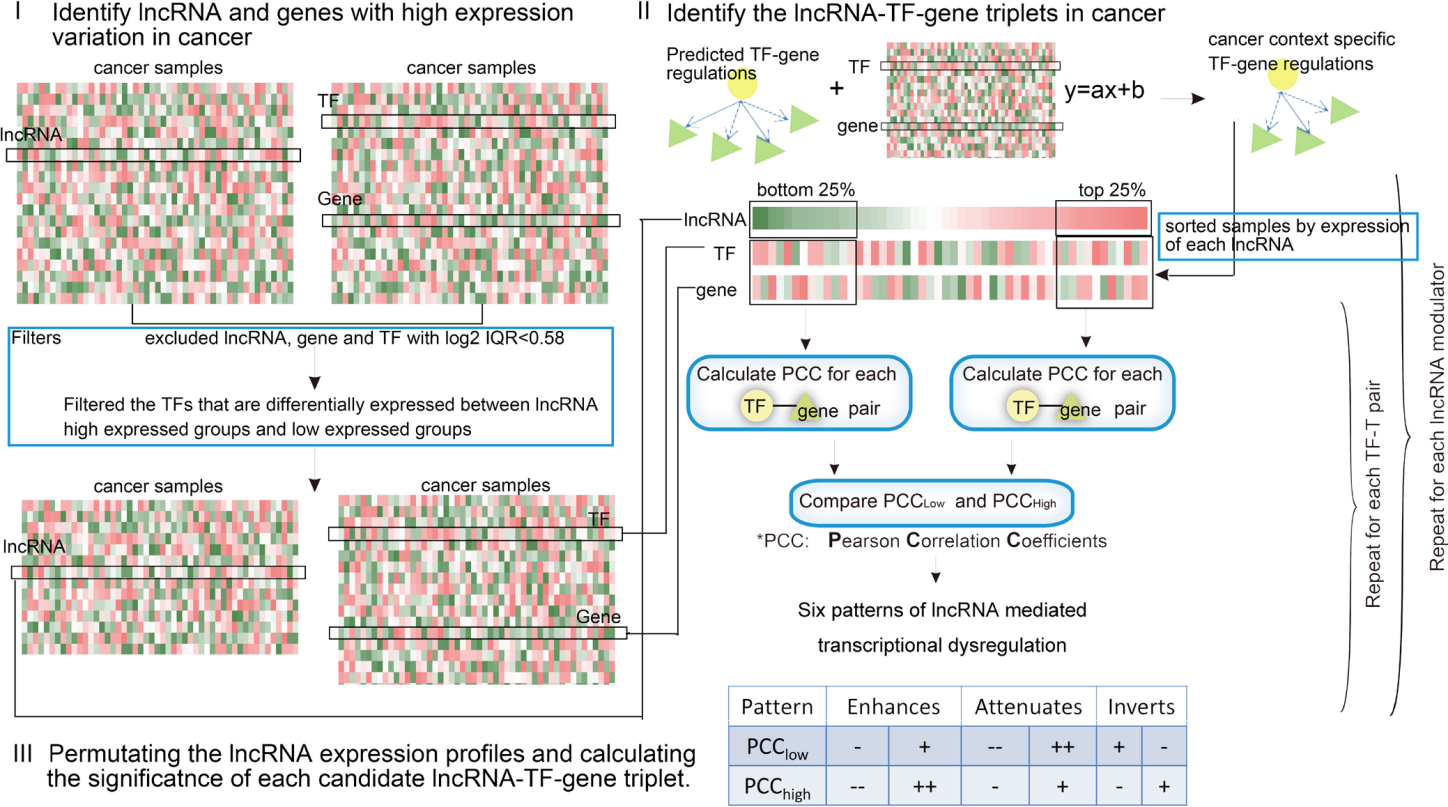
Schematic overview of the identification of lncRNA modulators in cancer For each candidate lncRNA-TF-T triplet, individual lncRNA, TF, and gene were selected based on their variation across samples. The relationship between TF-gene was then determined to be altered or not in the presence/absence of a given modulator lncRNA. Finally, the expression of lncRNAs were permutated to obtain the significance of each triplet.

With the increased availability of large data sets derived from high-throughput experiments and computer algorithms, investigating complex transcriptional misregulations mediated by lncRNAs in complex diseases is now possible. High-throughput methods are urgently needed to identify lncRNA regulators that affect TF activity in cancers. In this study, a computational framework is provided to comprehensively identify dysregulated lncRNA-TF-gene triplets by combining both lncRNA and gene expression profiles with TF-target relationships (Figure 1). This method was applied to glioblastoma (GBM) datasets to identify cancer-relevant lncRNA-TF-gene triplets. Notably, lncRNAs primarily affected target gene expression in trans. We identified six different classes of transcription regulation action for each lncRNA-TF-gene triplet. Functional analysis of the targets implicated lncRNAs in the regulation of some hallmark cancer genes. Finally, we described examples of lncRNA-mediated transcriptional dysregulation in lncRNA-TF-gene triplets that were associated with GBM prognosis. The method is available as an R package. We expect that the integration of lncRNAs into transcriptional regulatory networks will further enhance our understanding of lncRNA functions and provide new insights regarding cancer classification, prognosis, and treatment.

## RESULTS

### Overview of lncRNA modulator identification in cancer

Here, we developed a framework called LncMod for identifying lncRNA modulators by integrating genome-wide gene expression profiles and transcription regulation data. This process involved several scoring and filtering steps, as illustrated in Figure 1 and described further in the Materials and Methods. Briefly, paired lncRNA and gene expression profiles for specific cancers were obtained, and the lncRNAs, TFs, and genes were filtered based on the expression variation across samples (‘range constraint’). In addition, the expression of the candidate lncRNA modulators and TFs were required to be statistically independent (‘independence constraint’). The estimator then assessed the statistical significance of differences in correlation (measured by Pearson Correlation Coefficient, PCC) between the TF and a target in two subsets: the top and bottom 25% of samples in which the candidate lncRNA modulator is most and least expressed. The 25% parameter was determined empirically in previous study [18]. Each possible lncRNA-TF-gene triplet was independently tested using the permutation method. False positives were controlled using appropriate statistical thresholds. Six possible modes of lncRNA action were identified, depending on whether the TF-target correlation increased or decreased as a function of lncRNA modulator expression. The proposed method used four inputs: the gene expression profile dataset for lncRNAs, TFs, and target genes, and context-specific TF-gene regulations. For each TF-gene regulation, we reported the associated lncRNA modulators along with their mode of action.

### Inferring misregulatory lncRNA-TF-gene triplets in GBM

**Figure 2:**
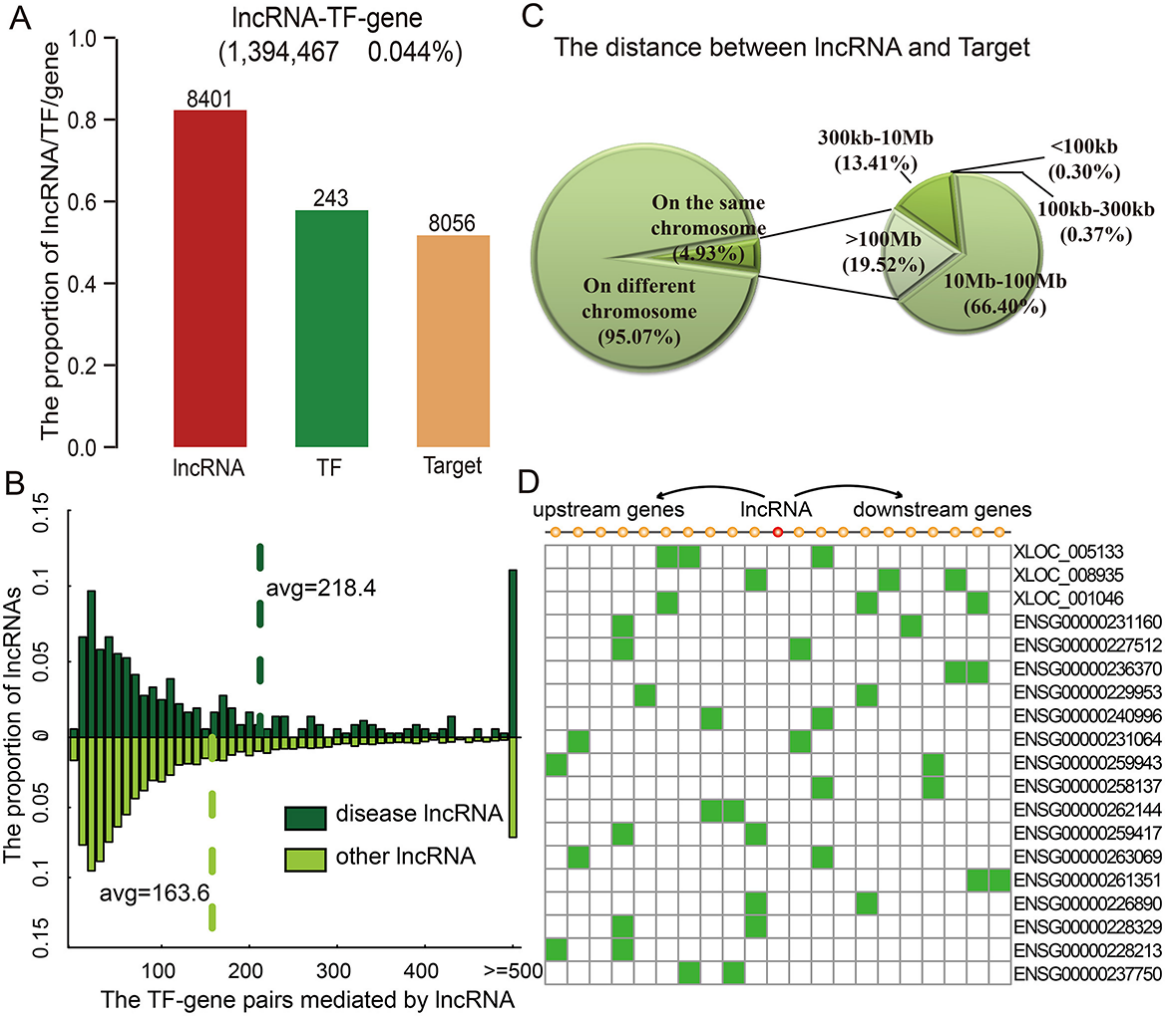
lncRNAs modulate transcriptional dysregulation in trans (**A**) The number of lncRNAs, TFs, and genes in the identified triplets. (**B**) Disease lncRNAs modulate more transcriptional dysregulations. (**C**) The distribution of distances between lncRNAs and genes. (**D**) Examples of lncRNAs that affect 10 neighbour genes on each side.

LncMod interrogated a large paired lncRNA and gene expression profile dataset to identify ‘lncRNA modulators’ whose expression strongly correlated with changes in the transcriptional activity of a TF. The statistical significance of changes in TF transcriptional activity (Δ*R*) can be effectively estimated from a large number of samples, provided that matched lncRNA and gene expression profiles are available for the same samples. We applied the proposed method for genome-wide identification of lncRNA modulators of TFs, using a previously assembled collection of GBM expression profiles from The Cancer Genome Atlas (TCGA). LncMod identified 8,401 lncRNA modulators participating in ∼139,000 lncRNA-mediated TF-target interactions at a conservative false discovery rate (FDR < 0.01, Figure 2A) in these GBM datasets. All of the identified triplets are enumerated in LncMod and are available online (http://www.bio-bigdata.com/LncMod/).

This global analysis produced two intriguing findings. First, lncRNA degree, defined as the number of transcriptional dysregulations it mediated, followed a power-law distribution with a slope of −1.284 and *R^2^* = 0.993 (Figure S1). The majority of lncRNAs mediated a low number of transcriptional dysregulations, while a few lncRNAs, termed hubs, mediated a high number of transcriptional dysregulations. KEGG enrichment analysis revealed that these target genes were enriched in categories known to be related to cancer development and progression (Figure S2), such as ‘focal adhesion’ (*p* = 6.0 × 10^−12^) and ‘P53 signalling pathway’ (*p* = 1.73 × 10^−5^). An early indication of the connection between the structure of a cellular network and its functional properties was the finding that highly connected proteins or hubs are more likely to be encoded by disease genes [1, 19]. This prompted us to hypothesize that human disease lncRNAs should also tend to mediate more transcriptional dysregulations. For example, the lncRNA HOTAIR mediated 513 transcriptional dysregulations among 71 TFs and 421 target genes. Our analysis showed that the number of transcriptional dysregulations mediated by disease lncRNAs was greater than that of other lncRNAs (Figure 2B, *P* = 6.22 × 10^−4^, Wilcoxon Rank-Sum Test). On average, disease lncRNAs mediated 218.4 TF-gene dysregulations, while other lncRNAs mediated approximately 163.6 dysregulations. The observed functional and topological centrality of lncRNAs fits well with our current understanding that many lncRNAs play critical roles in cellular development and growth.

### lncRNAs mainly affect TF activities in trans

lncRNAs may work either in cis or in trans to negatively or positively control protein-coding gene expression [20]. Next, we explored the distances between the lncRNAs and target genes in identified lncRNA-TF-gene triplets. LncRNA modulating the targets on the different chromosomes accounted for about 95.07% of triplets in GBM. Moreover, the majority of these lncRNAs seem to mediate transcriptional dysregulation in trans, more than 85.92% of these lncRNAs affected the transcriptional dysregulation of a gene beyond 10 Mb away (Figure 2C). A recent study concluded that lincRNAs act in cis based on the observation that knockdown of 7 out of 12 lincRNAs affected expression of a gene within 300 kb [21]. However, we found that only 0.67% of lncRNAs dysregulated the transcription of genes within this distance threshold in GBM. This is consistent with the observation of another recent study that only 8/147 lncRNAs affected genes within 300 kb; this proportion is lower than that observed for protein-coding genes [22]. However, we found that the proportions of lncRNA-gene pairs on the same chromosome and pairs further than 10 MB from each other were similar to randomly selected lncRNA-TF-gene triplets. For instance, HOTAIR represses transcription in trans across 40 kb of the HOXD cluster [23]. Here, we found that HOTAIR also mediated transcriptional dysregulation in trans. Just 6.41% of the target genes mediated by HOTAIR were on the same chromosome as it, and the distance between the nearest affected gene (ORMDL2) and HOTAIR was more than 1.8 Mb. Besides these trans-regulating lncRNAs, 402 lncRNAs in GBM affected genes located within 10 genes of the lncRNA in either direction, and only 19 lncRNAs affected more than two genes within this range (Figure 2D); these proportions are similar to those observed for randomly selected lncRNA-TF-gene triplets. For example, XLOC_008935 and XLOC_005133 only mediated the transcriptional dysregulation of three neighbor genes. In short, the majority of lncRNAs seem to affect the activity of TFs largely by acting in trans, but some also work in cis.

### Complex patterns of lncRNA-mediated transcriptional dysregulation

**Figure 3:**
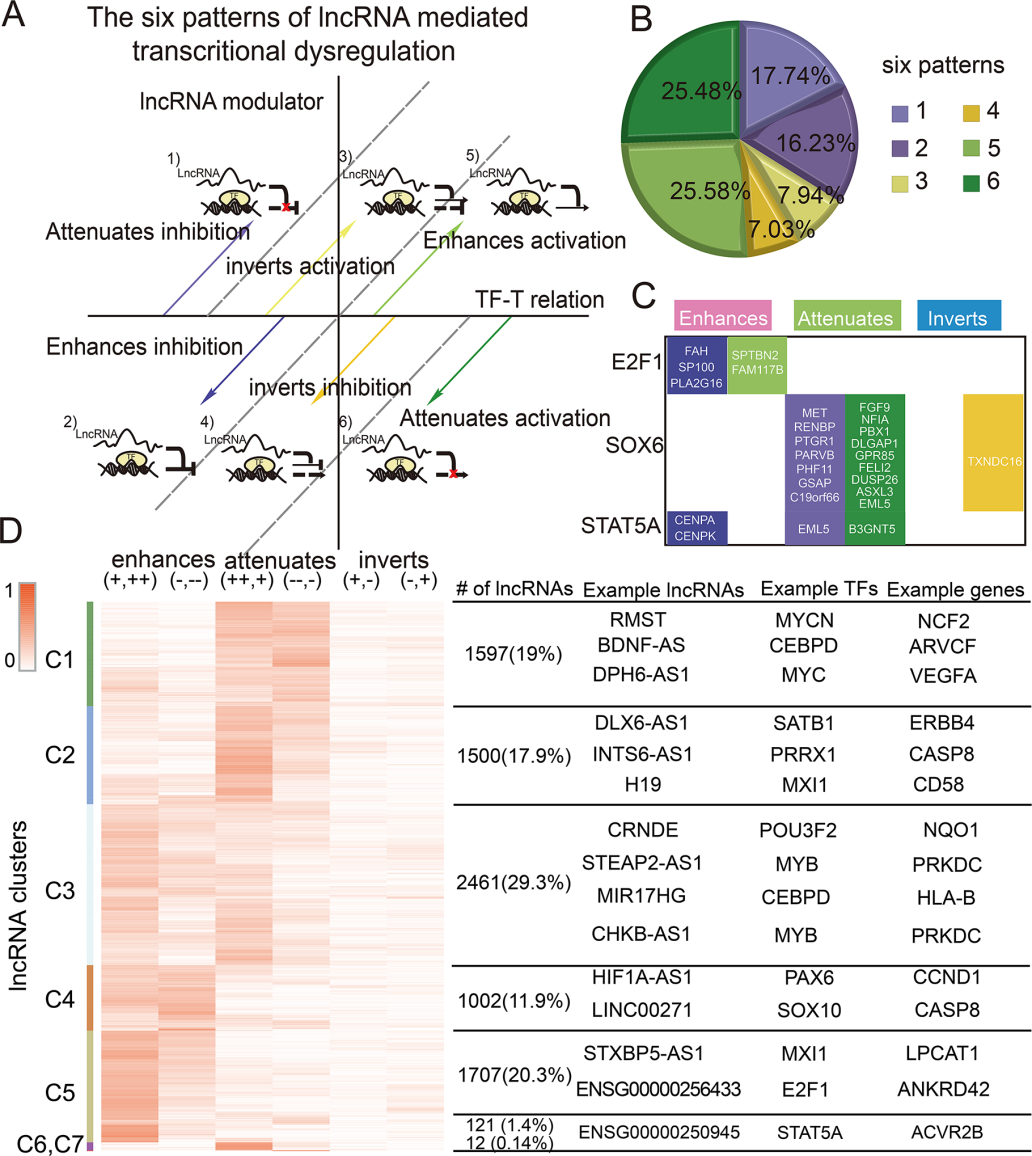
The complex patterns of lncRNA mediated transcriptional dysregulation (**A**) Classification of lncRNA modulators. (**B**) The proportion of each pattern. (**C**) Target genes of some TFs detected to be modulated by HOTAIR. (**D**) Clustering of lncRNA modulators based on regulation patterns. Some representative lncRNAs, TFs, and genes are shown on the right.

Many TFs both activate and repress gene expression depending on sequence, chromatin structure, and modulators. lncRNAs also affect specific subsets of TF targets, functioning as ‘coactivators’ or ‘corepressors’ [24]. In addition, lncRNAs may reverse the effect of TFs on target genes. The ternary lncRNA-TF-gene relationship is complicated. Genome-wide analysis of the lncRNA-TF-gene triplets in GBM showed that lncRNAs can not only enhance or attenuate the effects of TFs, but can also reverse them. To capture this complexity, we assigned each lncRNA-TF-gene triplet to one of six different patterns (Figure 3A). Globally, the majority of lncRNAs fine-tuned the expression of target genes in GBM. Approximately 85.03% lncRNAs enhanced or attenuated the effect of the TF in GBM, and 14.97% lncRNAs reversed the effect of TFs (Figure 3B).

In addition, we observed that most lncRNAs (98.94%) affected multiple genes and were multimodal; lncRNAs fit more than one pattern, acting as enhancers, attenuators, or invertors based on the target gene. For example, HOTAIR enhanced the association between STAT5 and CENPA, but also inhibited the association between STAT5 and EML5 in GBM (Figure 3C). It is well documented that gene modulators affect specific subsets of TF targets in different patterns [9]. Our findings support this complexity in that lncRNA modulators typically had many target-specific effects. These findings provide new insight on the role of lncRNAs in regulating the activity of TFs, and suggest that more complex models are needed to better elucidate how gene expression is regulated. lncRNAs were clustered by their regulation patterns, yielding distinct groups of lncRNAs that mediated transcriptional dysregulation in specific patterns (Figure 3D). For instance, the lncRNAs in cluster 4–5 tend to enhance TF activity, whereas those in cluster 1–2 tend to attenuate TF activity. These lncRNAs may function as coactivators or corepressors to mediate transcriptional regulation. However, clustering of lncRNA-mediated TFs showed that the majority of TFs and target genes were widely distributed in all lncRNA clusters (Figure S3). These observations indicated that the distinct lncRNA patterns were TF-gene-regulation dependent, but did not depend on specific TFs or target genes.

### lncRNAs modulate cancer-associated functions

Although accumulating evidence has provided insight into the various functions of lncRNAs, the exact functions of the majority of such transcripts are still unknown. In this study, we showed that lncRNAs mediated many transcriptional dysregulations. Investigating the functions of their target genes may provide new insights about the functions of these lncRNAs. One characteristic of cancer is the presence of abnormal cells that grow beyond their natural boundaries, and this property is driven by hallmark biomarkers of human cancers [25]. Although the biology of cancer is extremely complex, it can be well-represented by a few markers that enable tumor growth and metastasis dissemination. These “hallmarks” provide a framework for understanding may diverse types of cancer.

**Figure 4:**
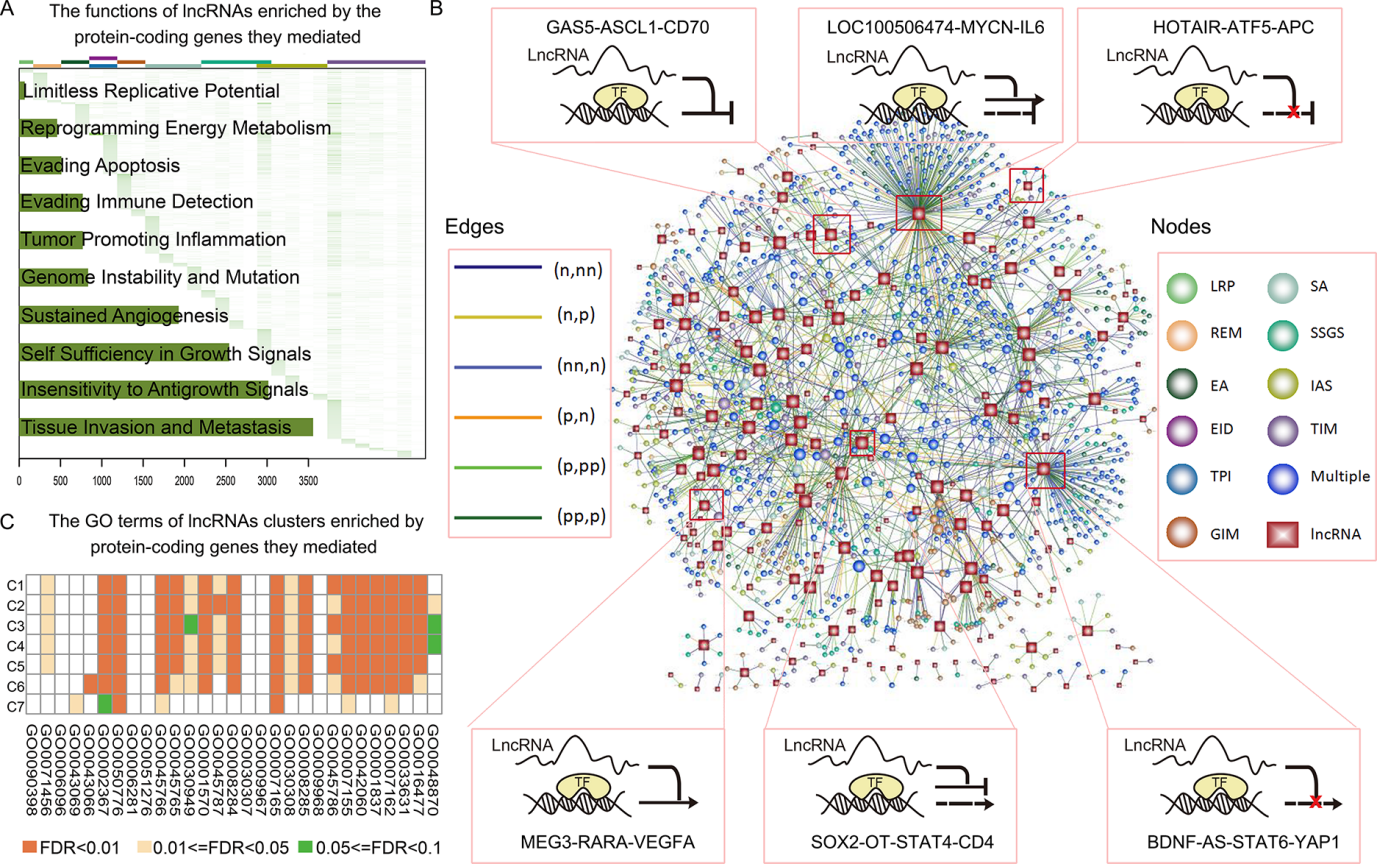
lncRNA modulated wide cancer-associated functions (**A**) Heatmap of lncRNA function profiles. Each row corresponds to a lncRNA and each column corresponds to a GO term associated with 10 hallmarks of cancer. Color represents the −log_10_ (*p*-adjusted). The number of lncRNAs enriched in each hallmark is shown in the bar plot on the left of the heatmap. (**B**) The representative lncRNA modulated transcriptional dysregulation associated with cancer genes. lncRNA and TF-gene pairs are shown as nodes and the lncRNA was linked to the TF-gene if it mediated the transcriptional dysregulation. Rectangles, lncRNAs; circles, TF-gene pairs. Node colors indicate hallmarks and the edge colors indicate effect patterns. (**C**) The heatmap of the function profiles of seven lncRNA clusters. Color represents the –log_10_ (*p*-adjusted).

Cancer-relevant lncRNAs were identified through analysis of target genes for functional enrichment of one or more hallmarks of cancer. In total, targets of 5,967 lncRNAs in GBM were enriched in at least one hallmark (Figure 4A). Globally, most lncRNA modulators regulated targets involved in hallmarks of tissue invasion and metastasis and insensitivity to antigrowth signals. Specifically, 463 of the disease-related lncRNAs identified were functionally implicated in known physiological or pathological processes; 445 lncRNAs also mediated transcription dysregulation of cancer genes (Figure 4B). The lncRNA with the highest connectivity in the network, BDNF antisense RNA (BDNF-AS1), mediated 124 transcriptional dysregulations, including some well-known GBM associated genes, such as YAP1, ITGB2 [26] and CDKN1A [27]. YAP1 is widely expressed in human brain tumors and promotes glioblastoma growth [28]. In addition, BDNF regulates cell growth, differentiation, migration, and apoptosis in the nervous system [29]. These observations suggest that novel therapeutic strategies that target BDNF might improve GBM treatment. A second lncRNA with high connectivity, LOC100506474, inverted the association between MYCN and IL6; recurrent amplification of LOC100506474 has also been demonstrated in GBM [30]. The lncRNA SOX2-OT, which mediated 22 pairs of TF-cancer gene dysregulations (Figure 4B), is highly expressed in tumors and is associated with the development of Alzheimer's disease [31]. Additionally, we showed that SOX2-OT inverted the transcriptional association of STAT4 to CD4, which plays a key role in cancers. These observations suggest that SOX2-OT may be a novel tumor therapy candidate. In addition, we found that MEG3 enhanced the positive regulation between RARA and VEGFA, and GAS5 enhanced the negative regulation between ASCL1 and CD70 (Figure 4B). These two lncRNAs play key roles in cancer development [32, 33]. Moreover, we examined the functions of distinct lncRNA clusters identified above and found that the functional profiles of distinct clusters were similar (Figure 4C). This suggests that more complex models and classification systems are needed to better elucidate how transcriptional regulation is mediated by lncRNA.

### lncRNA-TF-gene triplet signatures predict survival in GBM

Exhaustive survival analysis was also performed on each of the triplets in GBM to test whether their expression profiles were associated with cancer prognosis (details in Methods). Specifically, we focused on candidate lncRNA modulators that were located in amplified/deleted regions or have been previously associated with diseases. Studies suggest that genes with causal roles in oncogenesis are often located in the SCNAs (somatic copy-number alterations) that are frequently altered across tumors. To determine which lncRNA modulators might have tumor-promoting or -suppressing functions, we identified 239/168 lncRNAs in GBM that map to regions of recurrent amplification/deletion, respectively. It has been reported that some genes within amplified (or deleted) regions show increased (or decreased) expression levels that alter activity in cancer cells. We therefore reasoned that alternations in lncRNA modulator activity may dysregulate associations between TFs and their target genes. The functional importance of an lncRNA was only evaluated if one of its targets interacted with at least one of the cancer hallmarks. 9,359 triplets, consisting of 264 lncRNA modulators, 205 TFs, and 1,125 target genes, were identified for further analysis. Among these candidate triplets, 214 can be used to train and test GBM patients into good and poor prognosis groups (Figure S4). Although most lncRNAs in these triplets fine-tuned TF activity, TF activity was inverted in approximately 14.95% triplets. Additionally, although the combination of lncRNA, TF, and gene expression successfully stratified patients, approximately 97.20% of the triplets included a component that was not significantly associated with GBM prognosis. This suggests that studying dysregulation patterns at a cellular network level, rather than in a ‘gene-centric’ manner, may be a more efficient method of identifying prognosis biomarkers.

**Figure 5:**
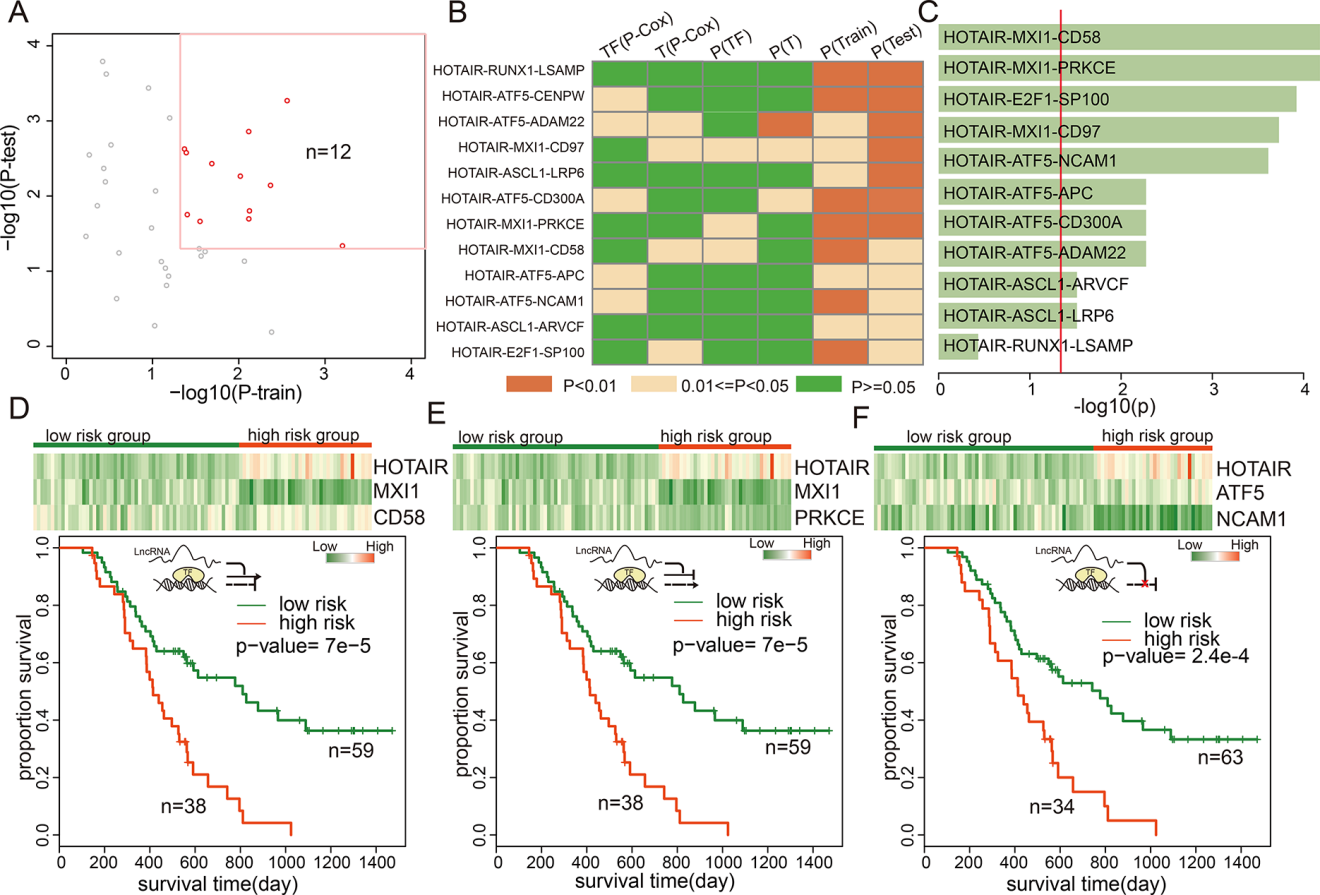
lncRNA triplets were associated with GBM prognosis (**A**) The prognosistic value of triplets with HOTAIR in training and testing datasets. (**B**) the *p*-values of the TFs, genes, or the triplets in training and test datasets. (**C**) the *p*-values of the lncRNA-TF-gene triplets involving HOTAIR in the validation dataset. (**D**–**F**) Color-gram of lncRNA-TF-gene expression profiles of GBM patients in the independent dataset. Rows represent lncRNAs, TFs, or genes and columns represent patients. The green (red) color bar represents low-risk (high-risk) patient groups. (Bottom panel) Kaplan–Meier estimates of overall survival of GBM patients according to the triplet signature.

As an example, 12 triplets involving HOTAIR were identified as being associated with GBM patient survival (Figure 5A). Of these 12 triplets, HOTAIR attenuated transcriptional regulations between five TF-gene pairs, enhanced regulation of five TF-gene pairs, and inverted regulation of two TF-gene pairs. In addition, our data suggested that HOTAIR was a negative prognostic factor in GBM (beta = 0.11, *p* = 0.043, univariate Cox regression in the train dataset). HOTAIR inverted the activity of MXI1 on CD58. Using combined HOTAIR-MXI1-CD58 expression, patients in the training set were divided into high- and low-risk groups; patients with high-risk scores had shorter median survival than those with low-risk scores (Figure S5, *p* = 0.008). Next, we conducted a test in which samples were also classified into high or low-risk groups using the same cut-off points as in the training set to validate this triplet signature. Again, patients with high-risk scores had shorter overall survival (Figure S5, *p* = 0.016). MXI1 over-expression inhibits the proliferation of U87 GBM cells, and MXI1-deficient mice show increased tumorigenesis [34]. In addition, activated CD58 upregulates the Wnt pathway, and knockdown of CD58 impairs sphere formation and tumor growth [35]. In a second triplet, HOTAIR-MXI1-PRKCE, HOTAIR also inverts the activity of MXI1. The PRKCE kinase is involved in many different cellular functions, such as neuron channel activation, apoptosis, cardioprotection from ischemia, heat shock response, and insulin exocytosis. Survival analysis revealed that combined HOTAIR-MXI1-PRKCE expression successfully stratified patients into good and poor prognosis groups in both the training and testing datasets (Figure S6). However, the expression of MXI1 and PRKEC could not distinguish patients based on survival times (Figure 5B, Cox regression *p* > 0.05), suggesting that the ‘triplet biomarkers’ are more informative than individual genes. Moreover, TF-ATF5 activity was also mediated by HOTAIR, which supressed negative regulation between ATF5 and NCAM1. ATF5 is essential in the genesis of malignant glioma [36], and analysis of human malignant glioma samples indicated that ATF5 expression inversely correlated with disease prognosis. NCAM1 protein is involved in the development of the nervous system [37], and in cells involved in T cell and dendritic cell expansion, which plays an important role in immune surveillance. Transcriptional dysregulation mediated by HOTAIR may serve as new targets for the diagnosis, therapy and prognosis in GBM.

To confirm the lncRNA-TF-gene triplet signatures as independent predictors, we obtained expression data for HOTAIR and the genes from an independent cohort of 97 Chinese GBM patients [38]. The K-means clustering procedure was used to divide patients into two subgroups based on triplet expression, and the Kaplan-Meier method was then used to estimate overall survival time for the two subgroups. Differences in survival times were analyzed using the log rank test. Expression of 11 of the 12 triplets modulated by HOTAIR was measured in this dataset. The expression of 10 (90.90%) triplets was associated with survival in the Chinese GBM patients (Figure 5C and Figures S5–S14). In addition, we observed that the three triplets discussed above yielded low *p*-values in the validation dataset (Figure 5D–5F). This suggests that HOTAIR plays an important role in GBM molecular classification and may serve as a novel therapeutic target.

## DISCUSSION

Here, we introduce LncMod, a new computational method for the identification of lncRNA modulators affecting TF activity in cancer. By integrating genome-wide lncRNA and gene expression profiles with TF-target regulation, LncMod identifies lncRNA modulators that affect TF activity, but not mRNA levels. By applying LncMod to published human GBM-associated lncRNA and gene expression datasets, we demonstrated that lncRNAs mainly affect target gene expression in trans. In addition, we found that most lncRNAs affect multiple targets and are multimodal, acting as enhancers, attenuators, or invertors depending on the specific target. Our results suggest that many more lncRNAs besides those identified in classical studies may affect cellular functions. In our study, functional analysis of these lncRNAs based the targets they regulate identified additional cancer-relevant lncRNAs. We focused on lncRNAs located in amplified or deleted regions and provided examples of lncRNA-TF-gene triplets (such as HOTAIR-MXI1-CD58/PRKCE and HOTAIR-ATF5-NCAM1/APC) that are associated with GBM prognosis. Kaplan–Meier survival curve analysis indicated that GBM patients with lower HOTAIR expression showed prolonged survival compared to patients with high HOTAIR levels (*p* < 0.001) [39]. In addition, we compared triplet signatures with previously identified prognosis-associated biomarkers in glioma, such as VSIG4 [40] and TRIM8 [41]. The *P*-values of the triplet signatures are much smaller than these previous identified biomarkers. Identification of triplets for which expression correlates with survival may improve our understanding of tumor development and provide more accurate information for the development of new targeted therapies.

**Figure 6:**
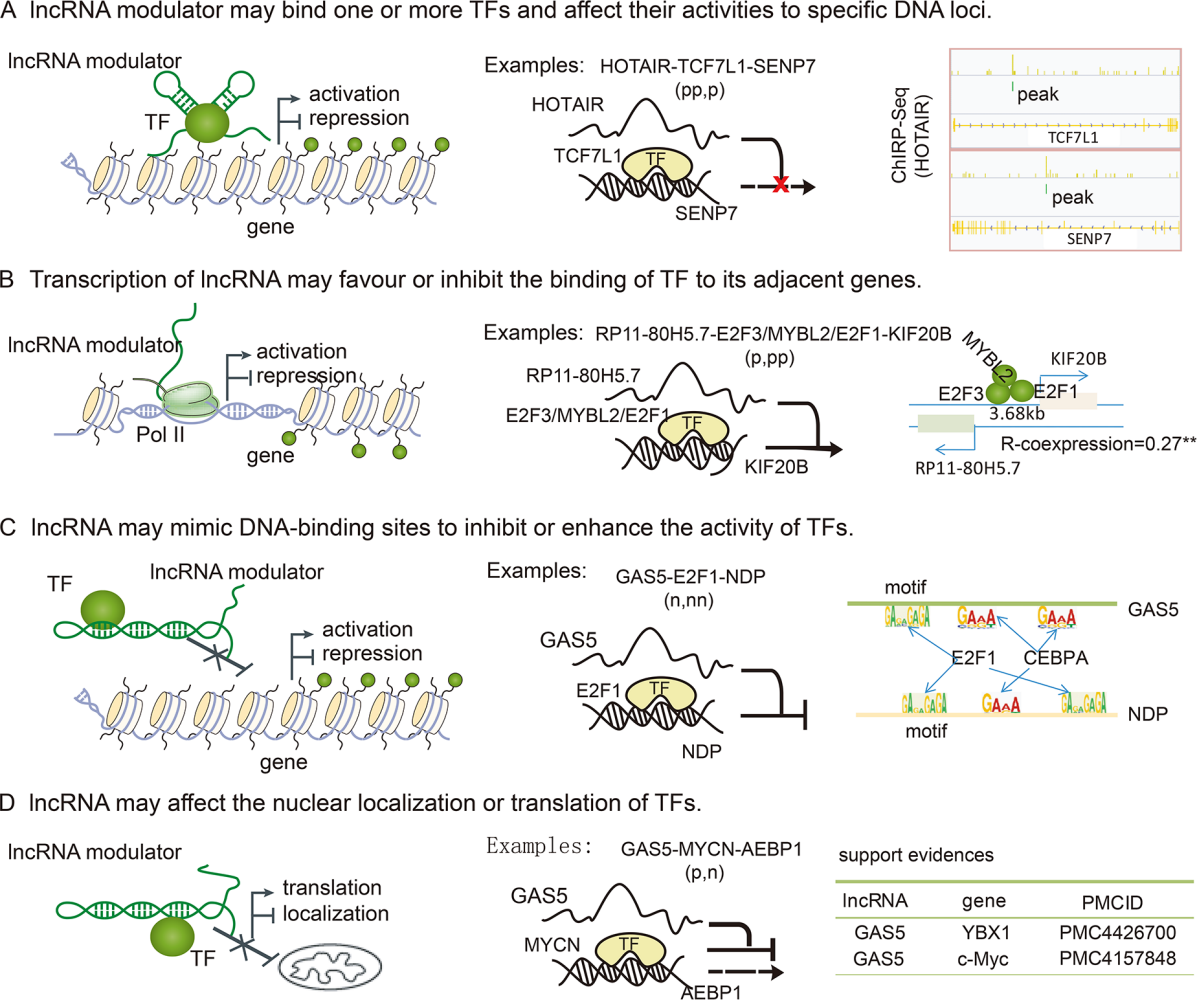
LncRNA mediated the activity of TF through several mechanisms (**A**) lncRNAs serve as scaffolds to bind TFs and target genes, and then affect the association of TFs with specific DNA loci. For example, HOTAIR can bind TCF7L1 and SENP7. (**B**) Transcription of lncRNAs can also result in chromatin remodelling that can either favor or inhibit the binding of TFs to their adjacent genes. Depending on the nature of TFs, gene expression is activated or repressed. (**C**) lncRNAs can fold into structures that mimic TF binding sites, and then inhibit or enhance the associations of TFs with their target genes. For example, the TFs mediated by GAS5 can also regulate the expression of GAS5, causing TF levels and activities to influence other genes. (**D**) lncRNAs can also regulate gene expression by binding TFs to inhibit the nuclear localization or translation of specific TFs.

Although thousands of lncRNA modulators were identified in our current study, the underlying mechanisms of how lncRNAs affect TF activity remains to be discovered. lncRNAs can act as scaffolds for several proteins tethered to a specific cellular compartment and thus guide recruitment of proteins to specific target genes [24, 42–44]. We proposed that some of these lncRNA modulators may also disturb the activity of TFs (Figure 6A). The development of high-throughput strategies, such as ChIRP, allows for unbiased discovery of RNA-bound DNA and proteins. In a ChIRP dataset of HOTAIR in cancer [45], there was a trend in which HOTAIR-mediated targets slightly overlapped with interacting genes (*p* = 0.09, hypergeometric test). HOTAIR also specifically attenuated the association of TCF7L1 to SENP7. Additionally, HOTAIR can also bind to TCG7L1 and SENP7, suggesting that HOTAIR may function as a scaffold that modifies TF activity (Figure 6A). In addition, lncRNA interaction data in LncRNADisease [46] showed that H19 can interact with E2F1 in the nervous system. In the present study, we found that H19 mediated the association of E2F1 with its targets. Moreover, the ability of MIR155HG to mediate the association of MYB with its targets was also supported by the LncRNADisease database. The increase in publicly available datasets of lncRNA annotations and interactions will provide new insights into lncRNA-mediated transcriptional dysregulation in cancer. In addition, lncRNA transcription can also result in chromatin remodeling that either favors or inhibits the binding of regulatory factors (Figure 6B). This may be the primary action of some cis-acting lncRNAs, such as RP11-80H5.7 and KIF20B. These two RNAs were located adjacent to each other (3.68 kb) and were co-expressed with each other (*R* = 0.27, *p* < 1.0 × 10^−32^). This implies that expression of this lncRNA may enhance the assembly of TFs at this genomic region and then promote the transcription of target genes. Moreover, lncRNAs may fold into structures that mimic the DNA-binding sites of the TFs, and the resulting interaction may inhibit or enhance the activity of specific TFs (Figure 6C). For example, the lncRNA GAS5 binds to the DNA-binding domain of the glucocorticoid receptor (GR) by acting as a decoy “glucocorticoid response element (GRE)”, and thus competes with DNA GREs for binding to the GR [47]. In our study, we found that GAS5 can also be targeted by the TFs it mediates, such as CEBPA, E2F1, and HOXB4. Yolanda et al. recently demonstrated that two p53-regulated lncRNAs are also required for efficient binding of p53 to some of its target genes, modulating the p53 transcriptional network and contributing to apoptosis in cancer [48]. These observations indicate that TFs and lncRNAs may establish positive regulatory feedback loops to regulate TF activity. In addition, new lncRNA-dependent mechanisms of protein translation control have been described [49]. This suggests that some lncRNAs may mediate the translation of TFs and also affect TF activity (Figure 6D). For instance, GAS5 mediated the activity of MYCN, which is a member of the MYC family. A recent study demonstrated a role for GAS5 lncRNA in translation regulation through its interactions with eIF4E and c-Myc mRNA [50]. In addition, Liu et al. have demonstrated that GAS5 enhances G1 cell cycle arrest via binding to YBX1, which regulates p21 expression in cancer [51]. Additional mechanisms by which lncRNAs mediate transcription likely have yet to be discovered.

Although the performance of LncMod is encouraging, integrating more source data is likely to improve its ability to detect cancer-relevant triplets. As a preliminary test, we combined TF binding profiles using a linear regression model to identify transcriptional regulations between TFs and genes. Advances in DNA sequencing technologies have led to the development of ChIP-seq (chromatin immunoprecipitation followed by DNA sequencing), which allows rapid and genome wide analysis of TF binding in cells [52]. Integrating these ChIP-Seq datasets may provide more accurate data regarding transcriptional regulations in cancer. Moreover, since lncRNAs are also regulated by TFs and play important roles in cancer, further investigation of the lncRNA-TF-lncRNA regulation loop would be useful. We also examined the effects of HOTAIR on the transcriptional regulation between MXI1 and CD58/PRKCE/CD97 using public ChIP-seq datasets. HOTAIR is overexpressed in the K562 cell line compared with GM12878 cells (Figure S15A). Consistent with our above analyses, MXI1 activity was altered in these two cell lines (Figure S15B–S15D). These results indicated that, at the level of “epigenomic control”, HOTAIR altered the association between TF and its target genes. Currently, LncMod focuses on the transcriptional regulation of TFs in cancer, but modified algorithms based on the same principles could be applied for other regulations. Examples are already emerging in which lncRNAs act as competing endogenous RNA (ceRNAs) to mediate miRNA and mRNA regulation [53–55]. In addition to their roles in human development, lncRNA ceRNAs have been implicated in various cancers. In addition to in silico prediction strategies, recently developed high-throughput biochemical techniques (such as HITS-CLIP and PAR-CLIP) allow genome-wide identification of miRNA-lncRNA/mRNA regulations. Analyzing data obtained using these experimental techniques in a manner similar to that presented here will provide further insights into ceRNA regulation. Moreover, the increasing availability of sample-matched lncRNA and gene expression profiles may make it possible to generalize the models proposed here to other cancers.

We expect that the integration of lncRNAs into regulatory networks with help improve understanding of the transcriptional control of TFs. Here, we provide a large-scale survey of lncRNA modulators in GBM and speculate about transcriptional regulation based on this additional layer of RNA-based regulation. The lncRNA modulators identified here may offer new targets for cancer diagnosis and therapy and help improve prognoses.

## MATERIALS AND METHODS

### Paired gene and lncRNA expression profiles across cancers

We collected paired gene and lncRNA expression profiles for GBM (451 samples) from a recent study [30]. Briefly, the exon array data was downloaded from TCGA (https://tcga-data.nci.nih.gov/) and probe sets of Human Exon array were re-annotated to the human genome (hg19). lncRNA expression was calculated by summarizing the background-corrected intensity of all probes that were mapped to the gene. lncRNA expression was quantile normalized across different biological samples, and Combat was used to remove potential batch effects [56]. As a result, the expression of 10,207 lncRNAs and 18,319 protein coding genes were obtained for further analysis. All expression profiles were log2 transformed. In addition, we downloaded clinical annotations of GBM patients from TCGA.

### Collection of disease-associated lncRNAs and cancer genes

The LncRNADisease database curates experimentally supported lncRNA-disease association data [46]. We downloaded experimentally supported disease-associated lncRNAs from this database. Additional known disease-related lncRNAs were collected by manual curation of published literature. All of these known disease-related lncRNAs were re-annotated according to lncRNA genomic positions derived from the Ensemble database (http://www.ensembl.org/). Known disease-related lncRNAs were mapped to the lncRNA IDs in our current study when they had at least 80% reciprocal overlap. In total, 67 disease-related lncRNAs were identified. In addition, recurrent somatic copy-number alteration (SCNAs) regions in GBM were identified by the GISTIC method from a previous study [57]. SCNA magnitude was estimated as the log_2_ ratio of segmented copy numbers between cancer and control DNAs. lncRNAs were then mapped to these SCNA regions using bedtools; in total, 239 and 168 lncRNAs in GBM were located in the amplified and deleted regions, respectively. In total, 463 disease-associated lncRNAs were collected. In addition, we collected cancer-associated genes from public databases, including Cancer Gene Census (CGC, http://cancer.sanger.ac.uk/cosmic), Online Mendelian Inheritance in Man (OMIM) [58], and the Genetic Association Database (GAD, http://geneticassociationdb.nih.gov).

### Identification of TF-gene regulations

To identify the regulatory relationship between TFs and genes, we first downloaded the defined promoter region (−2000/+2000 bp around TSS) of the 32,941 RefSeq genes from the UCSC table browser. Then, we searched the binding sites of TFs using the MatchTM software that is integrated in TRANSFAC Professional (release 2013.6) [59]. We used pre-calculated cut-offs to minimize false positive matches (minFP) and create a high-quality matrix. To restrict the search, we required that TFs belong only to the human genome [12, 60].

Because physical binding of transcription factors is necessary, but not sufficient, for transcription initiation in the context of GBM, we used a linear regression model to obtain context-dependent TF-gene regulations in GBM. This process was performed using the ‘lm’ function of R programming tool in which gene expression (log_2_) changes as a linear function of a specific TF across tumor samples. The *p*-value computed for the linear regression model was corrected by the BH procedure, and only regulations with an adjusted *p*-value less than a predefined threshold were further considered. In our study, we chose the corresponding thresholds (*p* < 1.0 × 10^−10^) that resulted in passing *p*-values for the top 30% of regulations in GBM.

### Overview of the identification of lncRNA-mediated transcriptional dysregulations in cancer

Here, we pursued a framework called LncMod to identify the lncRNA modulators which affect TF activities in cancer by integrating genome-wide gene expression profiles and transcription regulations. Briefly, the ‘LncMod’ method is based on a multivariate statistical dependence model designed to capture a particular type of three-way interaction where the ability of a transcription factor, g_TF_, to control its target gene, g_t_, is influenced by a number of lncRNAs, which we call modulators (g_m_).

A flowchart outlining the identification of misregulatory lncRNA-TF-gene triplets in specific cancers is outlined in Figure 1. First, paired lncRNA and gene expression profiles for specific cancers were obtained, and lncRNAs, TFs, and genes were filtered based on the expression variation across samples (‘range constraint’). Individual TFs (g_TF_), target genes (g_t_) and lncRNA modulators (g_m_) were selected based on their variation across samples (log_2_ IQR > 0.58). In addition, the expressions of candidate lncRNA modulators and TFs were required to be statistically independent (‘independence constraint’). For each lncRNA g_m_, the tumor samples were then sorted based on the expression of g_m_; the top and bottom 25% of samples in terms of lncRNA expression were then contrasted. Downstream analysis was only performed on TFs that were deemed independent based on a lack of differential expression between the lncRNA high-expression and low-expression sample subsets at *p* < 0.01 and >1.5-fold changes using a standard *t*-test. Each possible lncRNA-TF-gene triplet was then independently tested to determine whether the relationship between the TF and the gene was altered in the presence/absence of a given lncRNA. The regulations were deemed altered if the difference between PCC_low_ and PCC_high_ was >Th1 and the absolute value of either PCC_low_ or PCC_high_ was > Th2. In our current analysis, Th1 and Th2 were chosen as 0.45 and 0.4 according to Heidi et al. [18].

To assess the statistical significance of the difference (Δ*R*) between PCC_low_ and PCC_high_, we generated a series of null hypotheses by measuring the Δ*R* distribution across random conditions. That is, for each (g_TF_, g_t_) gene pair, expression profiles in non-overlapping sample subsets that were used to measure the PCC_low_, PCC_high_, and Δ*R*, were chosen at random from the complete dataset, rather than based on the expression of a candidate lncRNA modulator. This process was repeated 100 times. The *p* value is the fraction of Δ*R* in random conditions that was larger than that in the real conditions; *p*-values were Bonferroni-corrected for the total number of candidate lncRNA-TF-gene triplets [61]. The triplets with adjusted *p*-values less than 0.01 were regarded as significant. The R source code for the calculation is available at http://www.bio-bigdata.com/LncMod/ or http://ftp.ctex.org/mirrors/CRAN/web/packages/LncMod/index.html.

### Category of lncRNA action

For each triplet (lncRNA, TF, target) identified above, we defined the mode of action of the modulator with respect to the the effect of TF on target. TFs can activate or inhibit the activity of target genes, and lncRNAs can enhance, attenuate, or invert the activity of the TF. In total, there are six possible categories of action. These cases and their interpretations are listed in Table 1.

**Table 1:** Categories of lncRNA meditated transcriptional regulations

Modulation category	PCC_low_	PCC_high_	D_PCC_
Attenuates inhibition	—	−	|PCC_high_| < |PCC_low_|
Enhances inhibition	−	—	|PCC_high_| > |PCC_low_|
Inverts inhibition	+	−	
Inverts activation	−	+	
Enhances activation	+	++	|PCC_high_| > |PCC_low_|
Attenuates activation	++	+	|PCC_high_| < |PCC_low_|

Note: ‘+’ and ‘−’ signs in the columns indicate positive and negative values of Pearson correlation coefficient, respectively.

### Functional analysis of lncRNA modulators

The identified lncRNA-TF-gene triplets serve as paradigms for understanding lncRNA functions. Function enrichment analysis was carried out via the targets of triplets to determine the functions of lncRNAs by a hypergeometric test. Specifically, a list of GO terms that were related to the hallmarks of cancer were obtained from a previous study [62] and genes annotated to these hallmark-associated GO terms were obtained from MsigDB V4.0, which is a collection of annotated gene sets for use with GSEA software [63]. The targets of TFs mediated by lncRNAs were used to identify the hallmarks related to lncRNAs. GO terms with adjusted *p*-values < 0.01 and including at least two genes of interest were considered associated with lncRNA modulators.

### Survival analysis

To identify the lncRNA-TF-gene triplets that could predict GBM patient survival, specimens were randomly assigned to a training dataset or a test dataset. Two sample subsets had the same number of patients. In the random assignment of patients, age and sex information was also considered to make sure that the training and test subsets were balanced with regard to these factors (Table S1). We then used univariate Cox regression analysis to evaluate the association between survival and the expression level of each lncRNA, TF, and gene. Regression coefficients with a plus sign indicated that increased expression was associated with decreased survival (risky factors), and, conversely, a minus sign indicated that increased expression was associated with increased survival (protective factors). A mathematical formula for survival prediction was then constructed, taking into account both the strength and direction for each factor in the triplet with respect to survival. As in one of our previous studies [38], the risk score for each patient *i* was calculated as follows:

$$\begin{array}{ll} \text{Risk}\_{\text{Score}}_{i}= & \alpha *Exp_{i}\left(lncRNA\right)\\ & +\beta *Exp_{i}\left(TF\right)\\ & +\gamma *Exp_{i}\left(\text{gene}\right) \end{array}$$

*α, β, γ* were the regression coefficients for lncRNA, TF, and gene in the training dataset, respectively. All patients in the training dataset were thus assigned to high-risk and low-risk groups using the median risk score as the cut-off point. Patients with higher risk scores were expected to have poor survival outcomes. The coefficient and threshold values derived from the training dataset were directly applied to expression data of the corresponding test dataset to divide the patients in the test dataset into high-risk and low-risk groups. The Kaplan-Meier method was used to estimate the overall survival time for the two subgroups, and differences in survival time were analyzed using the log rank test. All analyses were performed using R 2.13.2 statistical software.

## Supplementary Materials Figures and Table


