# Supplementary material for: Identification and characterization of lncRNA mediated transcriptional dysregulation dictates lncRNA roles in glioblastoma
Source: Oncotarget. 2016 Mar 1;7(29):45027–41. doi: 10.18632/oncotarget.7801 (PMC5216703; doi:10.18632/oncotarget.7801)
Supplement: Supplementary file 1 [file oncotarget-07-45027-s001.pdf]

Identification and characterization of lncRNA mediated transcriptional dysregulation dictates lncRNA roles in glioblastoma

Supplementary Materials

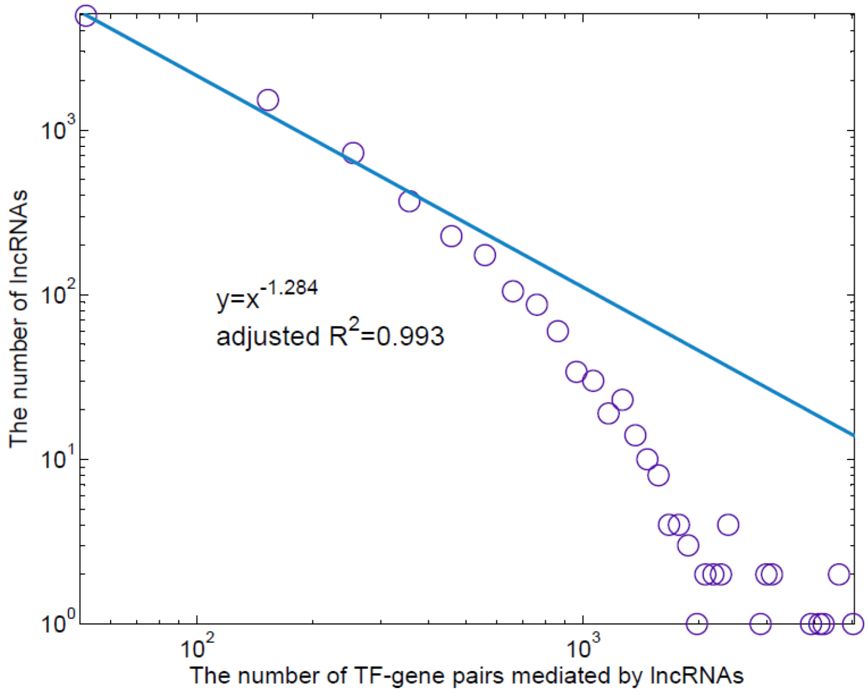

Supplementary Figure S1: The degree distribution of lncRNA modulators in GBM.

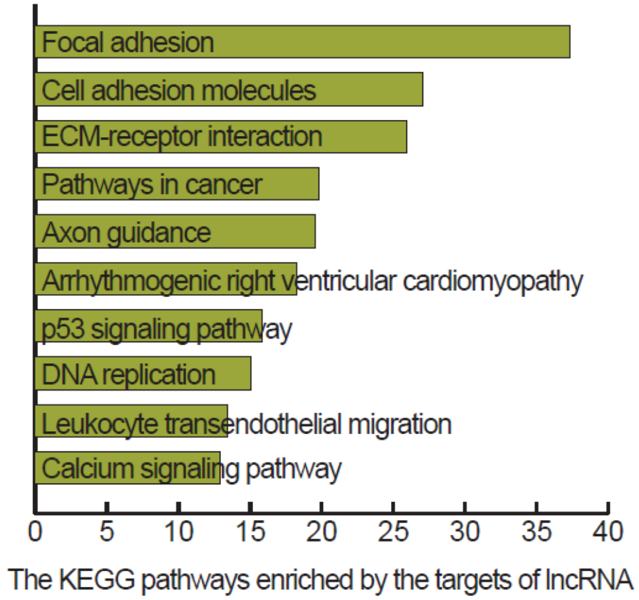

Supplementary Figure S2: The KEGG pathways enriched by the target genes of lncRNA modulators in GBM.

A The consistence of TFs among the seven lncRNA clusters

TF	cluster-1	cluster-2	cluster-3	cluster-4	cluster-5	cluster-6	cluster-7
cluster-1	1.0000	1.0000	0.9959	0.9876	0.9876	0.9008	0.3719
cluster-2	1.0000	1.0000	0.9959	0.9876	0.9876	0.9008	0.3719
cluster-3	0.9959	0.9959	1.0000	0.9835	0.9835	0.8971	0.3704
cluster-4	0.9876	0.9876	0.9835	1.0000	0.9917	0.9121	0.3766
cluster-5	0.9876	0.9876	0.9835	0.9917	1.0000	0.9121	0.3766
cluster-6	0.9008	0.9008	0.8971	0.9121	0.9121	1.0000	0.4128
cluster-7	0.3719	0.3719	0.3704	0.3766	0.3766	0.4128	1.0000

B The consistence of Targets among the seven lncRNA clusters

target	cluster-1	cluster-2	cluster-3	cluster-4	cluster-5	cluster-6	cluster-7
cluster-1	1.0000	0.9324	0.9416	0.9167	0.9377	0.4429	0.0453
cluster-2	0.9324	1.0000	0.9389	0.9092	0.9322	0.4443	0.0454
cluster-3	0.9416	0.9389	1.0000	0.9136	0.9436	0.4332	0.0444
cluster-4	0.9167	0.9092	0.9136	1.0000	0.9144	0.4544	0.0470
cluster-5	0.9377	0.9322	0.9436	0.9144	1.0000	0.4425	0.0453
cluster-6	0.4429	0.4443	0.4332	0.4544	0.4425	1.0000	0.0697
cluster-7	0.0453	0.0454	0.0444	0.0470	0.0453	0.0697	1.0000

C The consistence of TF-Targets among the seven lncRNA clusters (not considered the regulatory pattern)

TF-target	cluster-1	cluster-2	cluster-3	cluster-4	cluster-5	cluster-6	cluster-7
cluster-1	1.0000	0.5851	0.6015	0.4924	0.5565	0.0726	0.0046
cluster-2	0.5851	1.0000	0.5704	0.4743	0.5387	0.0849	0.0046
cluster-3	0.6015	0.5704	1.0000	0.5013	0.5745	0.0748	0.0042
cluster-4	0.4924	0.4743	0.5013	1.0000	0.4827	0.0831	0.0054
cluster-5	0.5565	0.5387	0.5745	0.4827	1.0000	0.0831	0.0043
cluster-6	0.0726	0.0849	0.0748	0.0831	0.0831	1.0000	0.0050
cluster-7	0.0046	0.0046	0.0042	0.0054	0.0043	0.0050	1.0000

D The consistence of TF-Targets among the seven lncRNA clusters (considered the regulatory pattern)

TF-target	cluster-1	cluster-2	cluster-3	cluster-4	cluster-5	cluster-6	cluster-7
cluster-1	1.0000	0.4222	0.3516	0.1842	0.2019	0.0446	0.0029
cluster-2	0.4222	1.0000	0.3457	0.2130	0.2127	0.0540	0.0026
cluster-3	0.3516	0.3457	1.0000	0.3173	0.3703	0.0355	0.0016
cluster-4	0.1842	0.2130	0.3173	1.0000	0.3410	0.0319	0.0012
cluster-5	0.2019	0.2127	0.3703	0.3410	1.0000	0.0251	0.0013
cluster-6	0.0446	0.0540	0.0355	0.0319	0.0251	1.0000	0.0029
cluster-7	0.0029	0.0026	0.0016	0.0012	0.0013	0.0029	1.0000

Supplementary Figure S3: The consistence of transcriptional dysregulations mediated by seven clusters of lncRNA modulators.

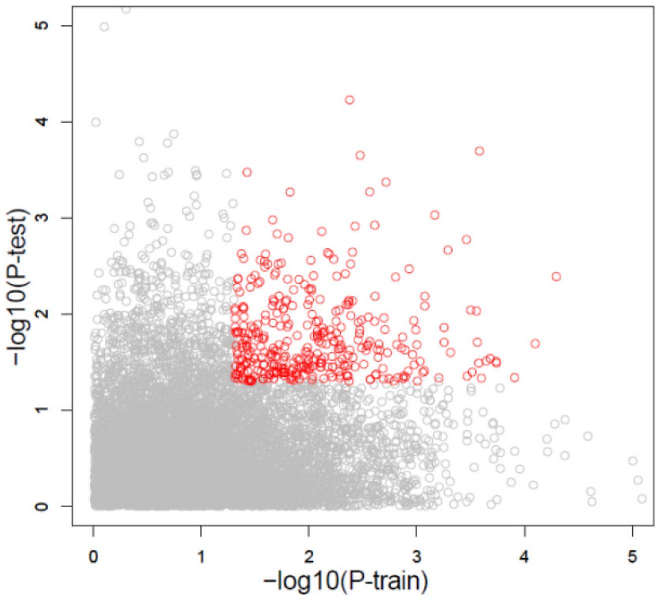

Supplementary Figure S4: The lncRNA-TF-target triplets associated with survival in the training and tesing datasets. The triplets (n = 214) with p less than 0.05 in both training and testing datasets were labeled in red.

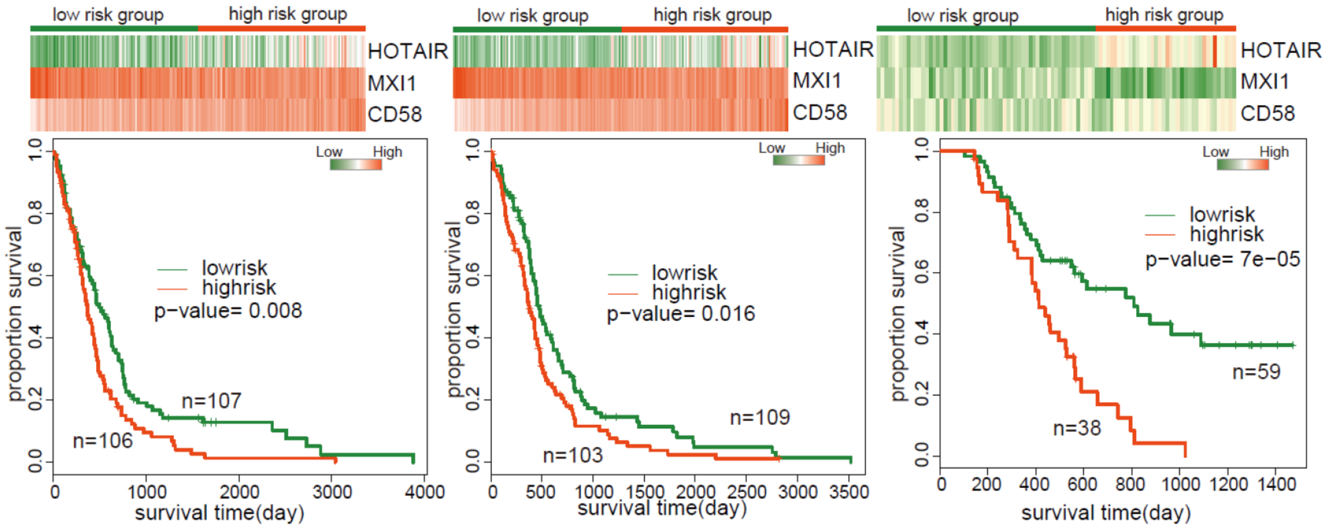

Supplementary Figure S5: The lncRNA triplet (HOTAIR-MXI1-CD58) was associated with the prognosis of GBM.

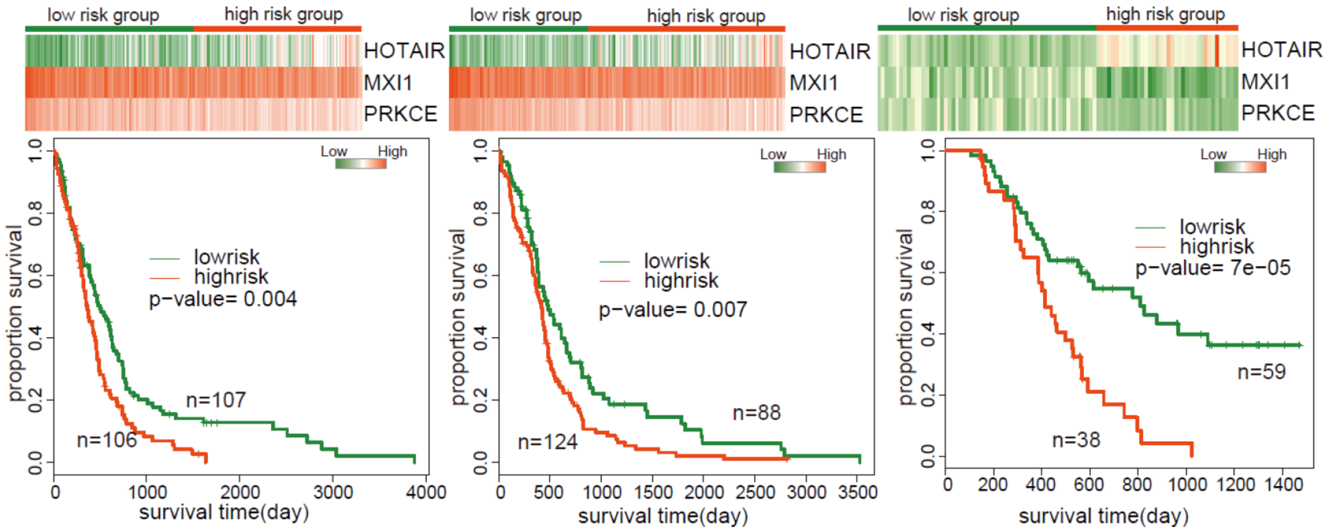

Supplementary Figure S6: The lncRNA triplet (HOTAIR-MXI1-PRKCE) was associated with the prognosis of GBM.

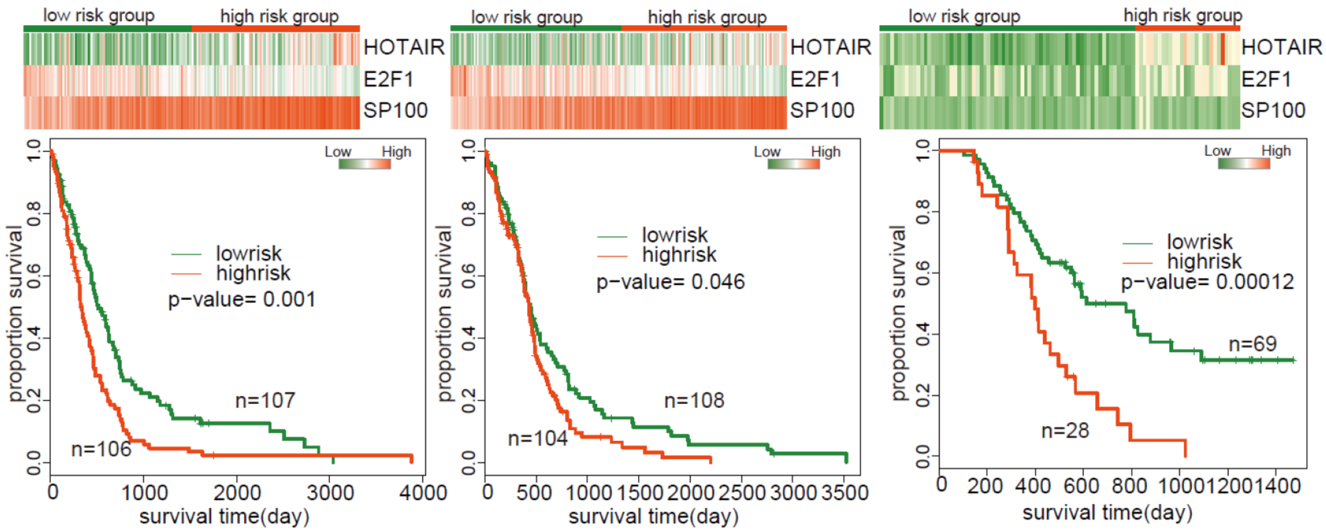

Supplementary Figure S7: The lncRNA triplet (HOTAIR-E2F1-SP100) was associated with the prognosis of GBM.

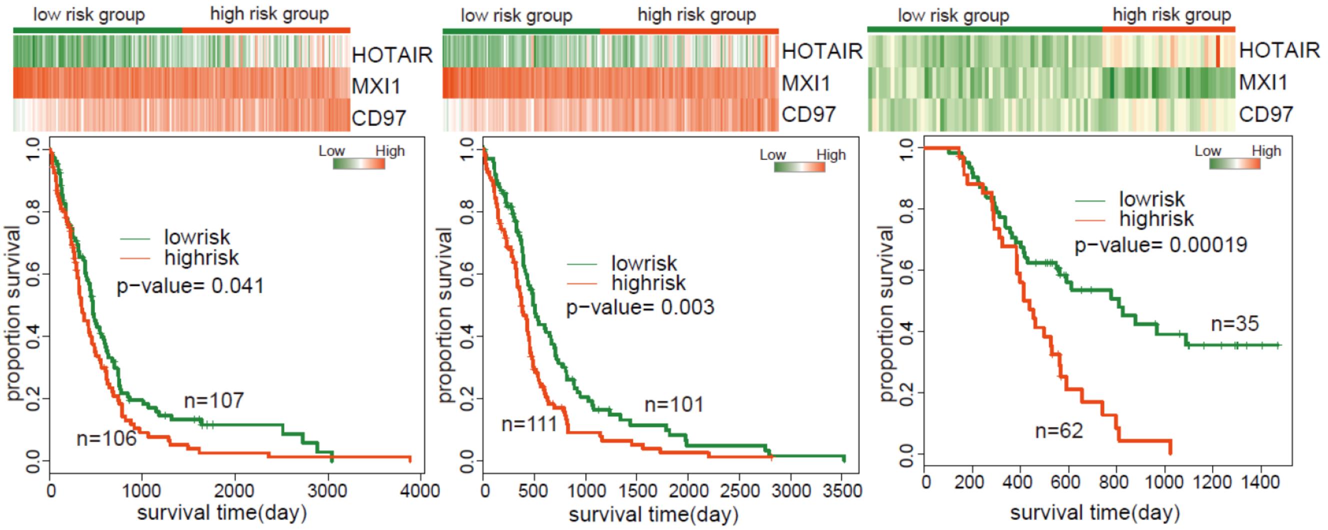

Supplementary Figure S8: The lncRNA triplet (HOTAIR-MXI1-CD97) was associated with the prognosis of GBM.

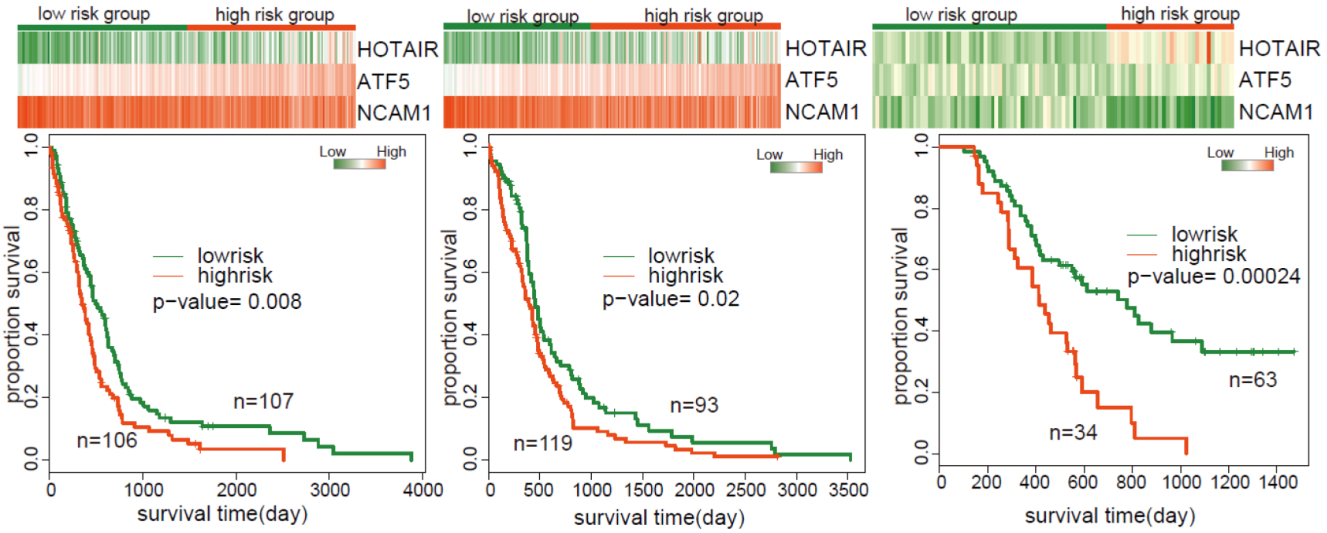

Supplementary Figure S9: The lncRNA triplet (HOTAIR-ATF5-NCAM1) was associated with the prognosis of GBM.

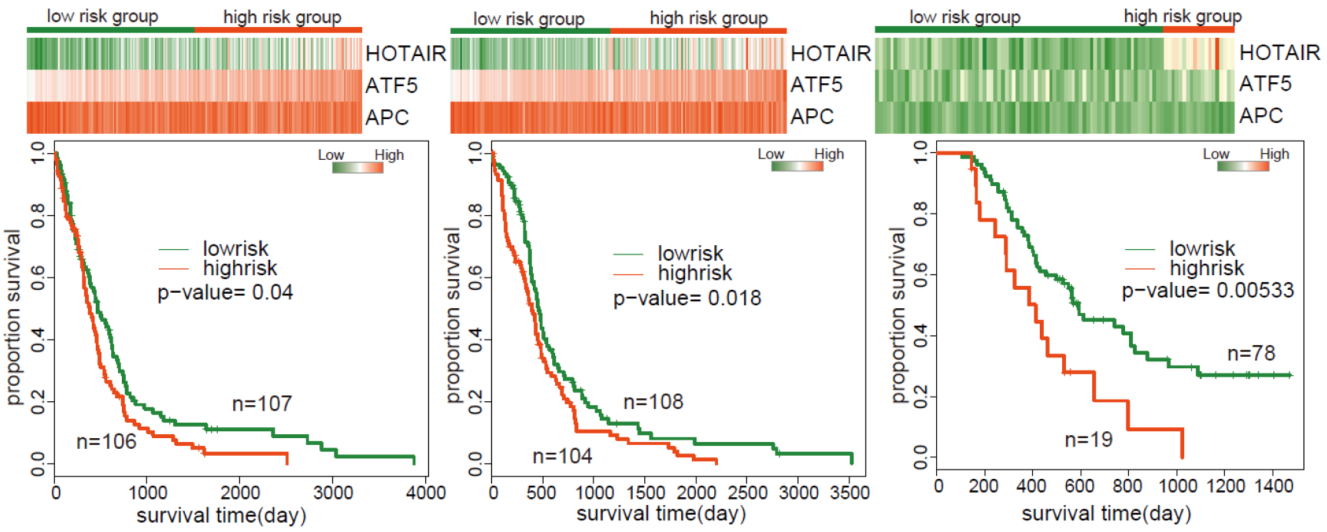

Supplementary Figure S10: The lncRNA triplet (HOTAIR-ATF5-APC) was associated with the prognosis of GBM.

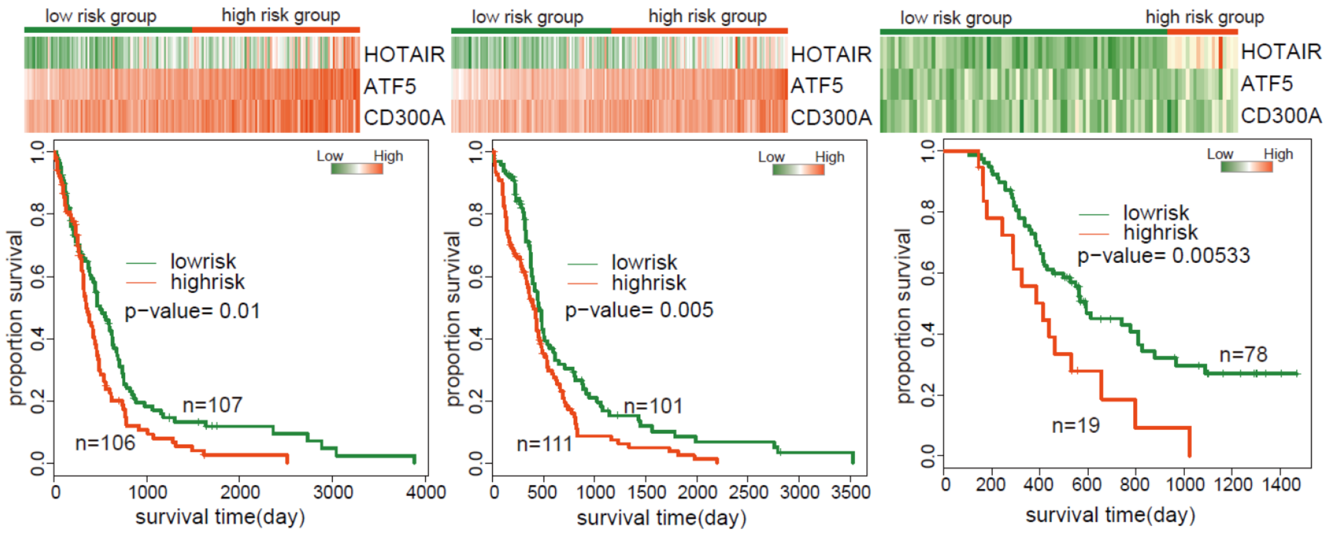

Supplementary Figure S11: The lncRNA triplet (HOTAIR-ATF5-CD300A) was associated with the prognosis of GBM.

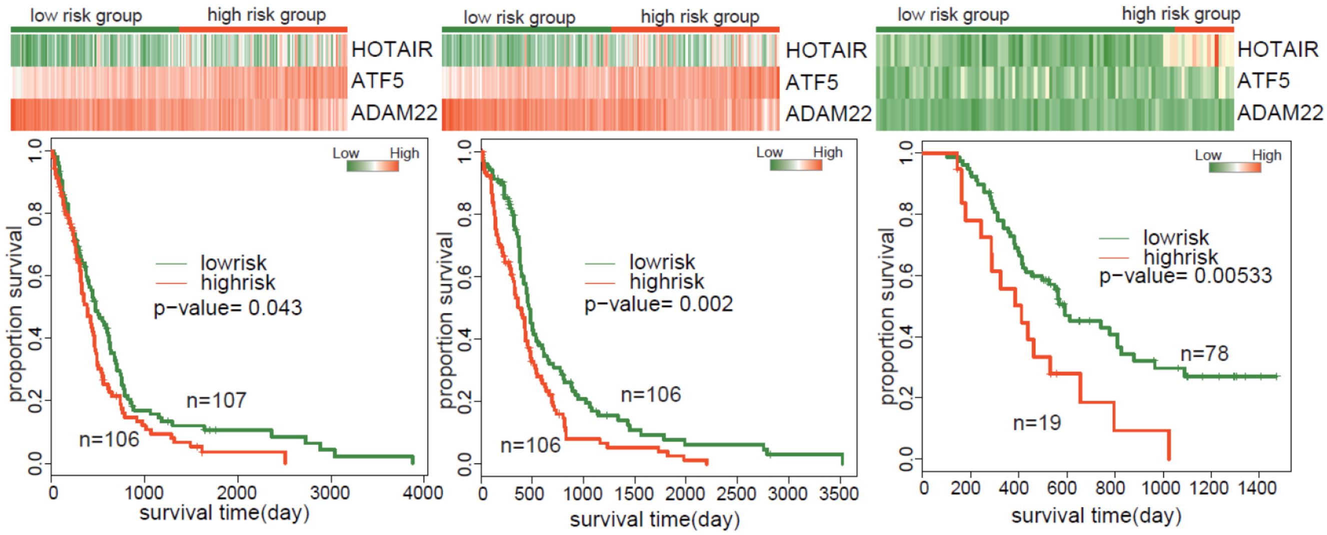

Supplementary Figure S12: The lncRNA triplet (HOTAIR-ATF5-ADAM22) was associated with the prognosis of GBM.

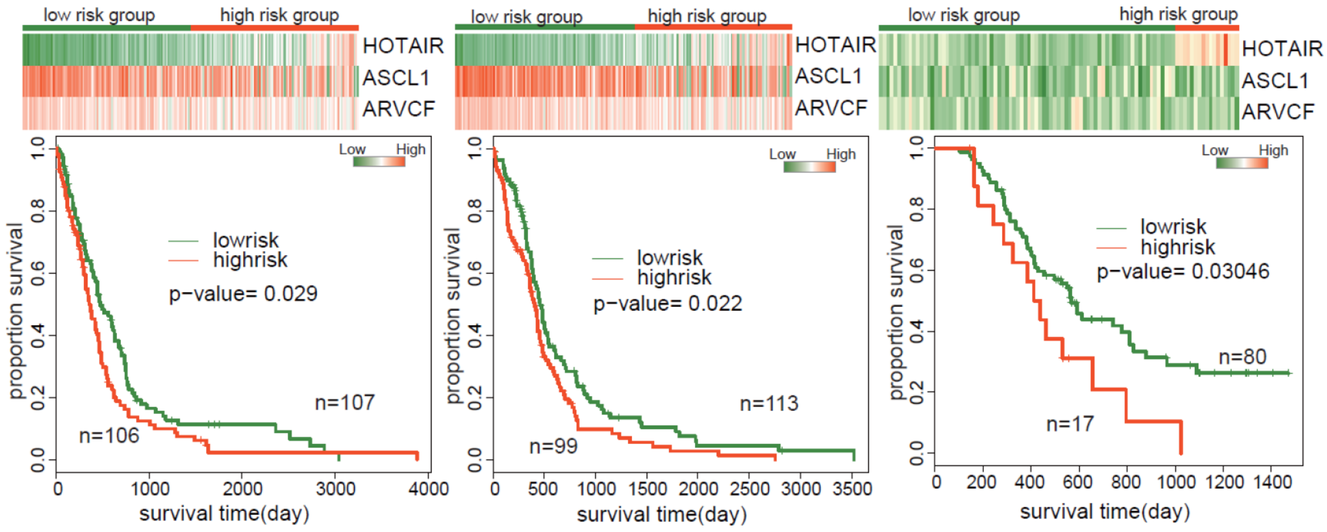

Supplementary Figure S13: The lncRNA triplet (HOTAIR-ASCL1-ARVCF) was associated with the prognosis of GBM.

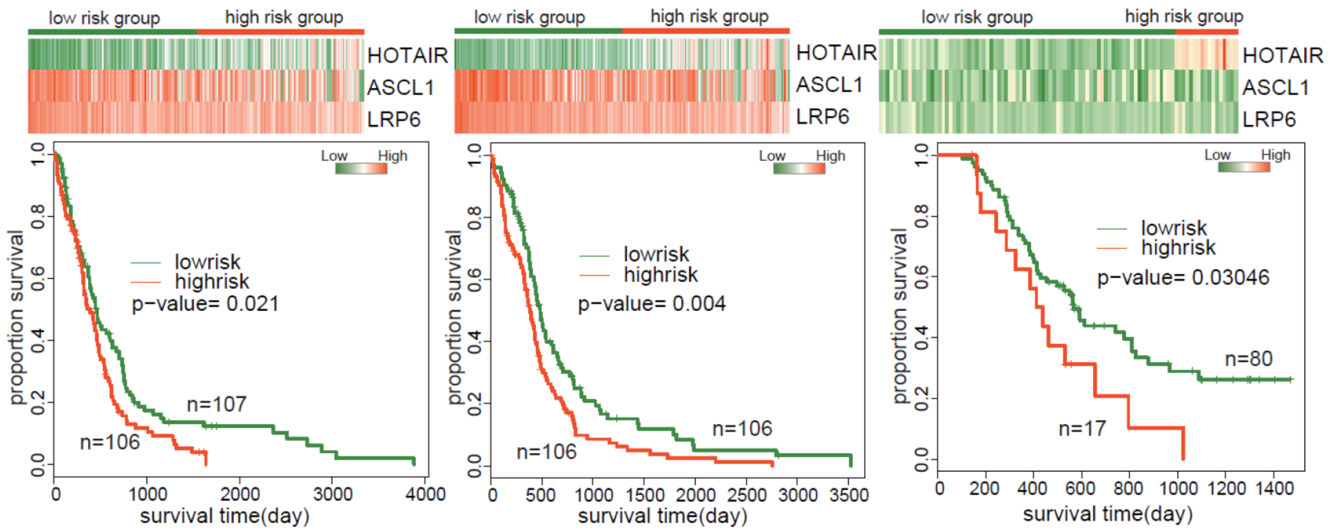

Supplementary Figure S14: The lncRNA triplet (HOTAIR-ASCL1-LRP6) was associated with the prognosis of GBM.

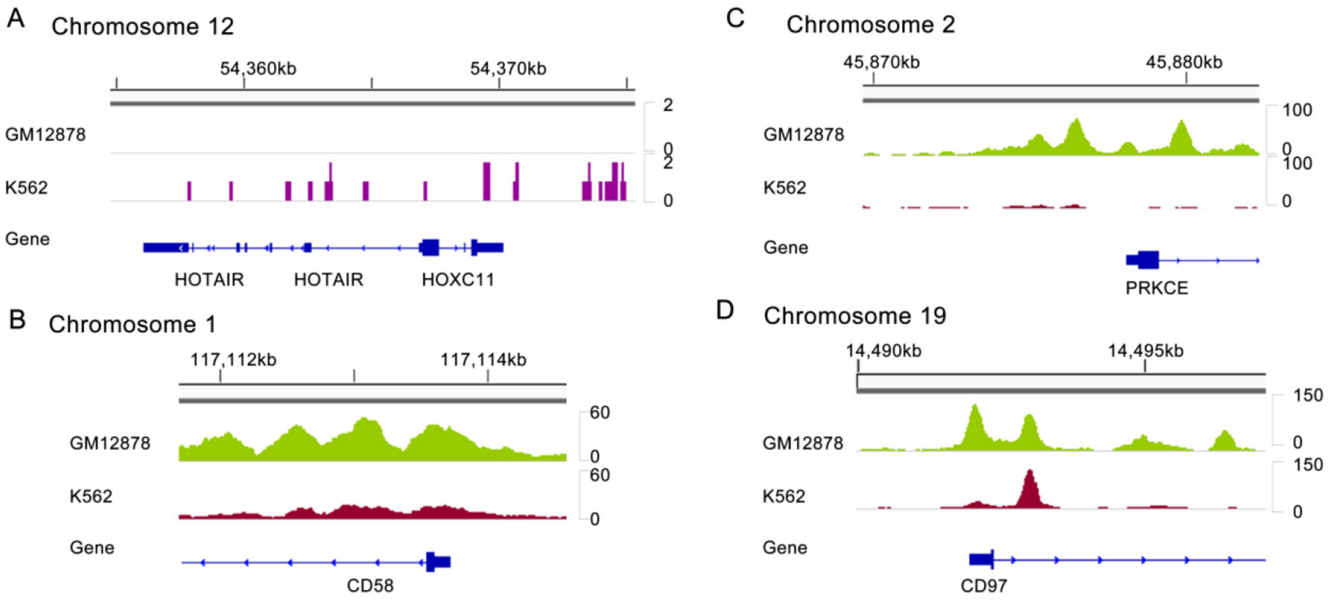

Supplementary Figure S15: The lncRNA HOTAIR alters the activity of MXI1. (**A**) the expression of HOTAIR in two cell lines. (**B**) the distribution of reads obtained by ChIP-seq (MXI1) in CD58 loci. (**C**) the distribution of reads obtained by ChIP-seq (MXI1) in PRKCE loci. (**D**) the distribution of reads obtained by ChIP-seq (MXI1) in CD97 loci.

Table S1: Clinicopathologic characteristics of patients with GBM

Cancer types	Characteristics	All patients	Number of patients		P
			Training set	Test set	
GBM (N = 431)	Sex				0.99[a]
	Female	160	80	80	
	Male	265	133	132	
	Age				0.84[b]
	Mean ± SD	57.00 ± 14.70	57.15 ± 15.28	56.87 ± 14.13	
	Range	10−89	10−89	14−88	
	Survival (month)				0.93[b]
	Mean ± SD	505.10 ± 537.51	502.70 ± 554.42	507.52 ± 521.28	
	Range	3−3880	8−3880	3−3524	
	State				0.70[a]
	Living	76	40	36	
	Death	349	173	176	
